# Functional profiling of the oral microbiome reveals microbial and oncogenic signatures in never-smoking female patients with oral squamous cell carcinoma

**DOI:** 10.1080/20002297.2025.2594842

**Published:** 2025-12-04

**Authors:** Sung Min Kim, Zeba Praveen, Yeon-Hee Kim, Jae Hee Ko, Yong-Seok Choi, Joo Yong Park, Jong Ho Lee, Sung Weon Choi, Mi Kyung Kim

**Affiliations:** a Oral Oncology Clinic, Research Institute & Hospital, National Cancer Center, Goyang-si, Gyeonggi-do, Republic of Korea; b Cancer Epidemiology Branch, Division of Cancer Epidemiology and Prevention, National Cancer Center, Goyang-si, Gyeonggi-do, Republic of Korea

**Keywords:** Oral squamous cell carcinoma, never-smoking females, oral microbiome, machine learning, *Rhodococcus*, LKB1, NF-kappa-B, PI3K-Akt signaling

## Abstract

**Background:**

The pathogenesis of oral squamous cell carcinoma (OSCC) in never-smoking females remains poorly understood, as these patients lack traditional risk factors. This subgroup accounts for an increasing proportion of OSCC cases and may exhibit distinct tumor biology. Here, we investigated the association between the alterations in the salivary microbiome and OSCC in never-smoking female patients.

**Materials and methods:**

Saliva samples from 72 never-smoking female patients with OSCC and 494 never-smoking healthy female controls were analyzed using 16S rRNA gene sequencing. Microbial community structure and function were compared using statistical analyses, machine learning algorithms, and pathway prediction with PICRUSt2.

**Results:**

Patients with OSCC exhibited significantly different microbial diversity and composition compared to controls. The genera *Rhodococcus*, *Slackia*, *Lactobacillus*, and *Enterobacterales_g* were enriched in the OSCC group, whereas *Corynebacterium* was more abundant in the Control group. These taxa were associated with oncogenic pathways, including PI3K-Akt signaling and nicotinate/nicotinamide metabolism. Functional inference also indicated enrichment of cancer-related orthologs such as *LKB1, NFKB1, ITGAV*, and *TRAF4*.

**Conclusions:**

Salivary microbiome alterations, both taxonomic and functional, are associated with OSCC in never-smoking females. These findings suggest a potential microbial contribution to carcinogenesis in this unique patient population and offer novel insights into disease mechanisms.

## Introduction

Oral cavity cancer is among the top 20 most common cancers worldwide, with an estimated 377,700 new cases and 177,700 deaths reported in 2020 [1]. Traditionally, the primary risk factors for oral squamous cell carcinoma (OSCC), a predominant histological type of oral cavity cancer, include tobacco use and alcohol consumption [2,3]. However, recent epidemiological data indicate a rising incidence of OSCC among women who have never smoked, a trend not fully explained by conventional etiologic pathways [4]. Tumors arising in never–smoking females may exhibit distinct biological characteristics, including more aggressive clinical behavior and poorer prognoses [5]. Consequently, conventional treatment strategies based on traditional risk factors or etiologic pathways may be inadequate for this subgroup, highlighting the need for novel, mechanism–based approaches. Therefore, a deeper understanding of the carcinogenic process in never–smoking females is crucial.

Recent studies have increasingly highlighted the critical role of chronic inflammation in the development and progression of various cancers, including OSCC. In the oral cavity, this can appear as periodontitis or gingivitis with signs such as bleeding and pocketing, and is marked molecularly by elevated cytokines (IL−6, IL−8, TNF-*α*), sustaining a tumor–promoting microenvironment [6–49]. Chronic inflammation can promote tumorigenesis through multiple mechanisms, such as inducing cytokine expression, suppressing anti–tumor immunity, and disrupting normal cellular signaling pathways [7]. Rather than being a mere response to tissue injury, persistent inflammation is now recognized as a key biological driver of malignant transformation by fostering genomic instability and creating a pro–tumorigenic microenvironment. In the oral cavity, this chronic inflammatory state can be observed clinically through conditions such as periodontitis and gingivitis, and is further supported by molecular evidence of elevated cytokines and microbial dysbiosis. In this context, factors that modulate chronic inflammation, such as the oral microbiome, have become key areas of investigation in cancer research [8–50].

The oral cavity harbors a complex microbial ecosystem that plays a critical role in modulating the immune system, sustaining chronic inflammation, and metabolizing carcinogens all of which are closely linked to tumorigenesis [6]. In particular, certain pathogenic microbes in the oral cavity can directly induce or sustain chronic inflammatory responses, thereby creating a tumor–promoting microenvironment [9]. Furthermore, Emerging evidence suggests that microbial dysbiosis, characterized by the overgrowth of pathogenic taxa and loss of commensal diversity, may contribute to both the initiation and progression of OSCC by sustaining chronic inflammation and modulating oncogenic signaling pathways [10]. Several studies have reported an enrichment of pathogenic species such as *Fusobacterium* within the oral microbiome of patients with OSCC, supporting its potential role in oral carcinogenesis [11]. However, most existing studies have focused on patients with OSCC with traditional risk factors or mixed cohorts, leaving the microbiome–associated oncogenic processes in never–smoking populations largely unexplored. Given that never–smoking female patients with OSCC exhibit distinct tumor biology and that microbial dysbiosis contributes to oral carcinogenesis, it is plausible that this subgroup harbors unique taxonomic and functional microbial alterations, independent of potential confounding effects from past smoking exposures.

To test this hypothesis, in the present study, we aimed to investigate oral microbiome composition and its oncogenic role in never–smoking female patients with OSCC, aiming to identify microbial biomarkers for early detection and prevention in this non–traditional risk group.

## Materials and methods

### Study population

In this case–control study, newly diagnosed female adult patients aged >19 years with histologically confirmed OSCC who had never smoked were recruited from the National Cancer Center, Goyang, Republic of Korea between 2016 and 2022. Tumors encompassed various anatomical sites within the oral cavity. Healthy female never–smoking individuals, matched by age, were selected as controls from the Cancer Screening Cohort of the National Cancer Center. All participants provided written informed consent, and the study was approved by the Institutional Review Board (IRB No. NCC2019−0050, IRB No. NCC2019−0116). Baseline oral health status was evaluated using panoramic radiographs and classified into stages I–IV according to the 2017 World Workshop classification of periodontal and peri–implant diseases (Table S6, Figure S2) [12].

### Saliva sample collection

Baseline saliva samples were collected from all participants following a standardized protocol [12]. Participants were instructed to refrain from eating, drinking (except water), brushing their teeth, or using mouthwash for at least one hour prior to sample collection. After the fasting period, participants were instructed to allow saliva to pool in their mouth and then passively drool into sterile 1.5 mL collection tubes until the required volume (approximately 1.5 mL) was obtained. Samples were immediately placed on ice, transported to the laboratory within 30 min of collection, and stored at −80 °C until further analysis. The saliva collection and processing adhered to established methodologies to ensure consistency and reproducibility [13,14].

### DNA extraction and 16S rRNA gene sequencing

Microbial DNA was extracted from saliva samples using the Fast DNA Spin extraction kit (MP Biomedical, Santa Ana, CA, USA) following the manufacturer's instructions. DNA quality and quantity were assessed using the Qubit dsDNA BR assay kit and a Qubit fluorometer (Life Technologies, Carlsbad, CA, USA).

The V4 region of the bacterial 16S rRNA gene was amplified using barcoded fusion primers 341F and 805 R (Bionics, Cosmogenetech, South Korea) [15]. The amplification was carried out using the following polymerase chain reaction (PCR) conditions: 95 °C for 3 min, followed by 25 cycles of 95 °C for 30 s, 55 °C for 30 s, and 72 °C for 30 s, and a final elongation at 72 °C for 5 min. PCR products were subjected to 2% agarose gel electrophoresis, and purified PCR products were used for secondary amplification to attach the Illumina NexTera barcodes. The reaction was carried out using Index 2 i5 forward and Index 1 i7 reverse primers (Bionics) with eight cycles of the aforementioned thermal cycling conditions, and the PCR products were purified using an AMPure bead kit (Agencourt Bioscience, Beverly, MA, USA). The amplicons were pooled using Chunlab (https://www.cjbioscience.com/) and sequenced on an Illumina iSeq100 platform at the National Cancer Center, South Korea. The 16S gene, which encodes the ribosomal RNA component, is commonly used for phylogenetic analysis due to its highly conserved nature and slow evolutionary rate [16]. Low–quality reads with (<80 bp or >2,000 bp) were filtered out using trimmomatic. DUDE–Seq was used for denoising and identification of non–redundant reads, and chimeric sequences were removed using the UCHIME algorithm against the Ezbiocloud 16s–Based Microbial taxonomic profiling database [15,17,18]. Taxonomic assignments were performed using USEARCH tools, and sequencing reads with 97% sequence similarity were classified into operational taxonomic units (OTUs) using the UPARSE algorithm [19,20]. The UCLUST tool was used to cluster single–end reads into OTUs based on predefined cutoffs [19].

### Functional profiling

Functional predictions were inferred using PICRUSt within the Microbiome Taxonomic Profiling pipeline in the EzBioCloud database [21]. Reads were obtained using the EzBioCloud 16S pipeline, then matched to reference database entries [17]. Functional profiles were annotated by multiplying gene counts per OTU with OTU abundance per sample using the KEGG database for pathways [22]. The nearest sequenced taxon index was used to assess inference accuracy.

### Statistical analyzes

Statistical analyzes were conducted using R version 4.1.1 (R Foundation for Statistical Computing, Vienna, Austria). Between–group comparisons of participant characteristics utilized t–tests and chi–square tests. Alpha diversity metrics (observed OTUs, Chao1, Shannon indices) and beta diversity (principal coordinates analysis [PCoA] based on weighted and unweighted UniFrac distances) were calculated (22). Microbial abundance differences were evaluated using Wilcoxon rank–sum tests, fold change analyzes, and linear discriminant analysis effect size (LEfSe) with linear discriminant analysis (LDA) score and cladogram visualizations (23). Logistic and multivariate–adjusted conditional logistic regression analyzes were also performed.

Machine learning analyzes were performed using Python 3.7.15 (Python Software Foundation) with the H2O module (version 3.38.0.2; https://github.com/h2oai/h2o−3). The Gradient One Side Sampling (GOSS) algorithm within the Light Gradient Boosting Machine (LightGBM) framework was implemented due to its computational efficiency, suitability for high–dimensional and imbalanced data, and integrated feature importance assessment. Model performance was evaluated using 5-fold cross–validation and external validation sets. Metrics assessed included accuracy, area under the curve (AUC), F1 score, precision, and recall. Hyperparameter tuning was conducted to optimize model performance, and the top 20 important features were extracted as potential diagnostic biomarkers.

To validate the relevance of oral microbiome–associated functional inferences, expression levels of cancer–related orthologs (LKB1, NF-κB, ITGAV, and TRAF4) were assessed using two pancreatic cancer microarray datasets–GSE16515 and GSE14245—from the Gene Expression Omnibus (GEO) database [23,24]. Although these datasets are not specific to oral cancer, they were used to explore whether genes predicted as functionally relevant in our OSCC cohort exhibit altered expression patterns in an independent cancer context. Expression data were compared using the Wilcoxon rank–sum test.

## Results

### Demographic characteristics of cases and controls

The clinical characteristics of the study participants are summarized in Table 1. A total of 72 never–smoking female patients with OSCC and 494 healthy female controls were included. Most patients with OSCC were above 60 years (61.1%), whereas a larger proportion of controls were below 60 years (64.6%). Moreover, the proportion of non–drinking (73.6%) was significantly higher in the OSCC group than that in the Control group (47.0). Regarding tumor staging, 50.0% of patients with OSCC were diagnosed with T3/T4 stage, while the remaining 50.0% presented with TNM stage T1/T2. Most patients (75.0%) were N0, indicating no regional lymph node metastasis at diagnosis. TNM staging of OSCC patients was determined according to the 8th edition of the AJCC (American Joint Committee on Cancer) and UICC (International Union Against Cancer) guidelines. The most common tumor subsites were the tongue (33.3%), followed by the lower gum (32.0%), upper gum (20.8%), buccal cheek (8.3%), and retromolar trigone (5.6%).

**Table 1.** General characteristics of 72 never–smoking females patients with oral squamous cell carcinoma (OSCC) and 494 control females in this study.

Characteristics		OSCC (*N* = 72)	Control (*N* = 494)	*P–value*
Age	≤60	28 (38.9)	319	<0.001
	>60	44 (61.1)	175	
BMI		23.99 ± 4.43	23.32 ± 3.46	0.144
Drinking	Drinking	19 (26.4)	262 (53.0)	0.002
	Non–Drinking	53 (73.6)	232 (47.0)	
TNM	Early (I–II)	29 (40.3)		
	Late (III–VI)	43 (56.7)		
T stage	T1, T2	36 (50.0)		
	T3, T4	36 (50.0)		
*N* stage	N0	54 (75.0)		
	N+	18 (25.0)		
Grade	PD	20 (27.8)		
	MD	32 (44.4)		
	WD	07 (9.70)		
	Missing	13 (18.1)		
Tumor subsite	Tongue	24 (33.3)		
	Buccal cheek	6 (8.3)		
	Upper gum	15 (20.8)		
	Lower gum	23 (32.0)		
	Retromolar trigone	4 (5.6)		

General patient characteristics were compared between OSCC and control groups using t–tests for continuous variables (Age and BMI) and chi–square tests for categorical variables (e.g. Drinking status, TNM stage, T stage, N stage, Grade, Tumor subsite). Drinking status was categorized into two groups: Drinking and Non–Drinking.

### Altered diversity and structure of the oral microbiota in never–smoking female patients with OSCC

**Figure 1.**
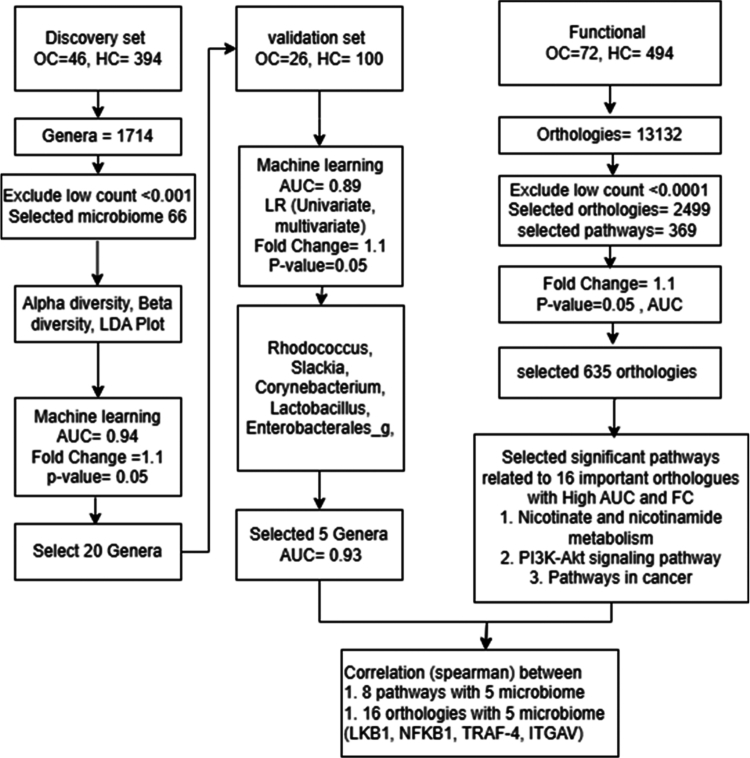
Utilization of advanced analytical techniques in the study**.** Overview of analytical strategies used in this study, including machine learning, correlation analysis, microbiome profiling, and functional profiling. Abbreviations: HC, never–smoking healthy control; OC, never–smoking female patients with oral squamous cell carcinoma; FC, Fold change; AUC; Area under curve; LR, Logistic Regression; LDA; Linear discriminant analysis.

**Figure 2.**
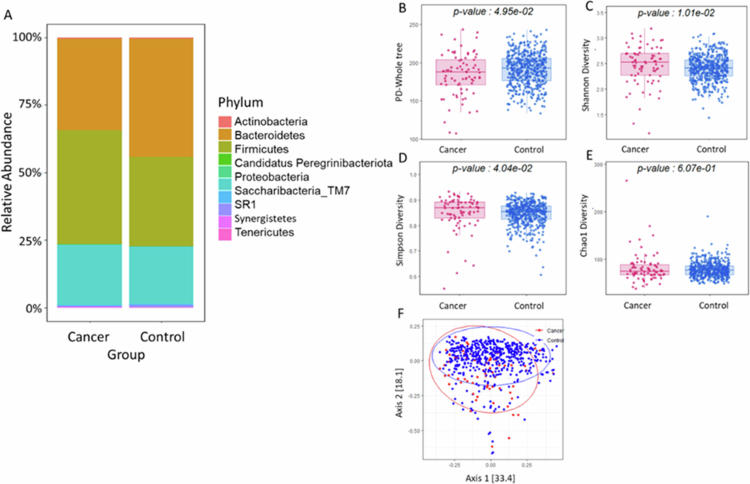
Comparison of oral microbial composition and diversity between never–smoking female patients with OSCC and healthy controls. (A) Average composition of the microbial community at the phylum level, showing taxa with a relative abundance greater than 1%. (B–E) Alpha diversity indices including (B) PD–Whole tree, (C) Shannon index, (D) Simpson index, and (E) Chao1 diversity are estimated. All showing significant differences (*P* < 0.001) in the OSCC group compared with Control group. (F) Beta diversity analysis using principal coordinate analysis (PCoA) based on weighted UniFrac distances reveals distinct clustering between never–smoking female patients with oral squamous cell carcinoma and healthy controls. OSCC, oral squamous cell carcinoma; PD, phylogenetic diversity; PCoA, principal coordinate analysis.

To investigate the diagnostic potential and biological relevance of the oral microbiome in never–smoking female patients with OSCC, we integrated microbiome profiling, machine learning classification, and functional pathway analysis (Figure 1). Comparison between the OSCC and Control groups revealed distinct microbial community structures. Across all samples, a total of 48 phyla and 1,714 genera were identified. At the phylum level, the dominant taxa included Bacteroidetes, Fusobacteria, and Proteobacteria, comprising over 90% of the bacterial population (Figure 2A). Alpha diversity analysis revealed mixed trends among indices (Figure 2B–E). The observed OTUs and Chao1 indices were significantly higher in OSCC patients, indicating increased richness in their microbial community (*p* < 0.05). Beta diversity analysis using weighted UniFrac distances demonstrated a significant difference in microbial community composition between the OSCC and Control groups (*p* < 0.001) (Figure 2F). These findings suggest that never–smoking female patients with OSCC exhibit altered oral microbial diversity and composition, potentially reflecting disease–associated dysbiosis.

### Machine learning–based identification of key oral microbiota associated with OSCC in never–smoking females

**Figure 3.**
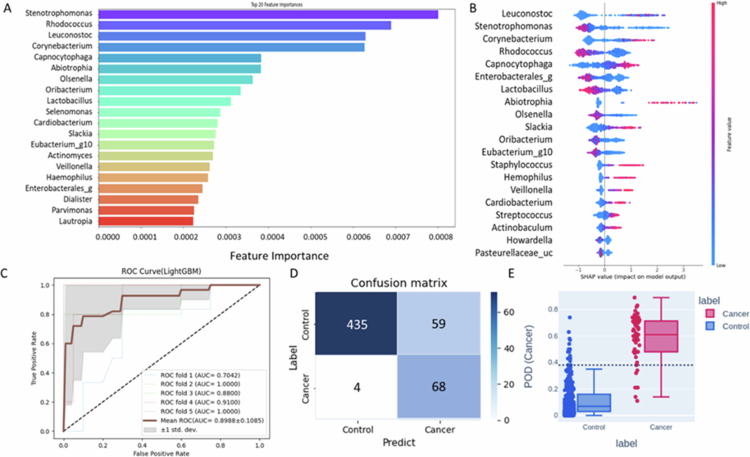
Oral microbiota–based machine learning model using Light Gradient Boosting Machine (LightGBM) with 5-fold cross–validation for classifying never–smoking female OSCC patients and healthy controls. (A) Feature importance analysis showing the top 20 features contributing to model performance in the discovery dataset. (B) SHapley Additive exPlanations (SHAP) values illustrating the contribution of each feature to individual predictions. (C) Receiver operating characteristic (ROC) curve analysis for model validation, illustrating true positive rate (TPR) vs. false positive rate (FPR). (D) Performance of LightGBM model evaluated using fold–wise confusion matrices derived from actual and predicted labels. (E) Probability of detection index (POD) generated using LightGBM, indicating the likelihood of classifying samples as cancerous (on a 0 to 1 scale).

**Figure 4.**
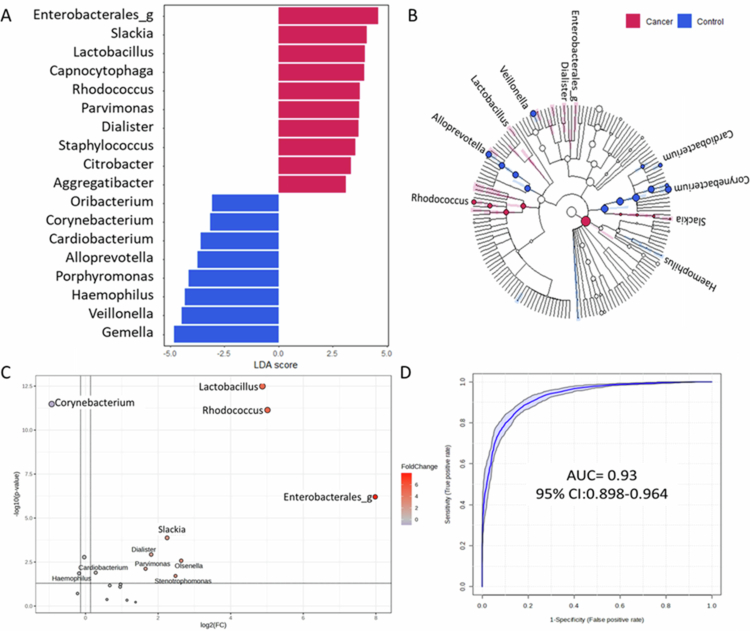
Differential analysis of oral microbiota between never–smoking female patients with OSCC and healthy controls. (A) Linear discriminant analysis effect size (LEfSe) analysis at the genus level. (B) Cladogram visualizing genus–level differences. (C) Volcano plot illustrating fold change analysis of 20 microbiomes, orthologies, and pathways between OSCC and Control groups. (D) Receiver operating characteristic (ROC) curve analysis of five selected genera (*Rhodococcus, Slackia, Lactobacillus*, *Enterobacterales_g, Corynebacterium*). Significant microbiome features enriched in never–smoking female patients with OSCC(red) and healthy controls (blue).

To identify microbial taxa associated with OSCC in never–smoking females, we employed a LightGBM–based machine learning approach. The model demonstrated robust performance, achieving an F1 score of 0.38, sensitivity of 0.88, precision of 0.86, accuracy of 0.82, and an AUC of 0.89 based on receiver operating characteristic (ROC) analysis (Figure 3A–E). LDA score at the genus level indicated a higher OSCC risk associated with *Rhodococcus, Slackia, Enterobacterales_g*, and *Lactobacillus*, while *Corynebacterium* was significantly associated with reduced risk (Figure 4A,B). Fold change analysis confirmed these patterns, with significant elevations in *Rhodococcus* (FC = 30.97, *p* = 2.71 × 10^−37^), *Slackia* (FC = 4.73, *p* = 2.34 × 10^−10^), *Enterobacterales_g* (FC = 45.57, *p* = 1.08 × 10^−22^), and *Lactobacillus* (FC = 109.64, *p* = 1.58 × 10^−27^) and significant depletion of *Corynebacterium* (FC = 0.54, *p* = 1.71 × 10^−36^) in the OSCC group. Genus–level logistic regression analysis further confirmed the discriminatory power of these taxa (Figure 4C, Tables S1–S2). Furthermore, a combined model incorporating these five genera yielded a strong diagnostic performance with an AUC of 0.93 (Figure 4D).

### Functional pathway shifts in the oral microbiome of never–smoking patients with OSCC

**Figure 5.**
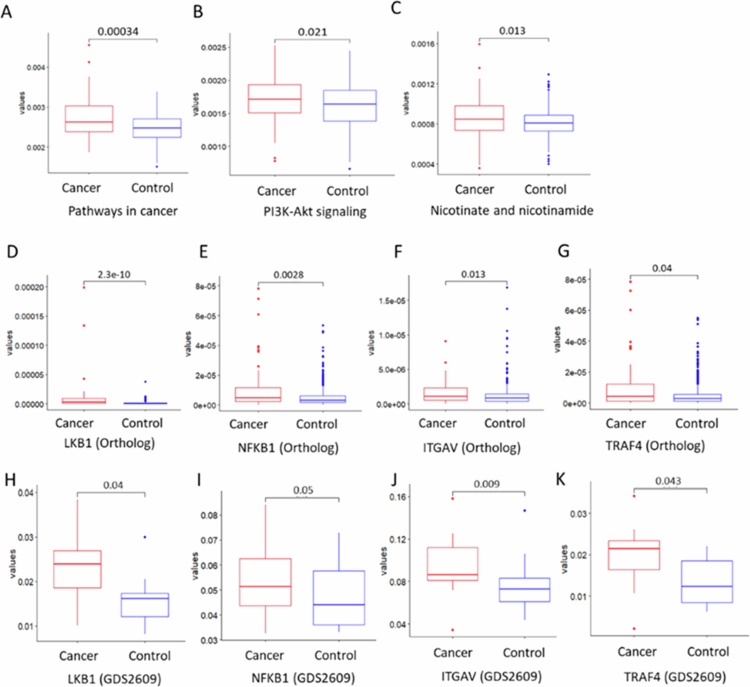
Functional profiling analysis comparing pathway abundances and key gene expression between never–smoking patients with oral squamous cell carcinoma (OSCC) and healthy controls. (A–C) Boxplots comparing pathway abundances between never smoking female patients with OSCC and healthy controls. (D–G) Boxplots showing the expression levels of key orthologues (*LKB1, NFKB1, ITGAV, TRAF4*) in the functional orthologies dataset. (H–K) Boxplots depicting expression profiling data from 96 tongue squamous cell carcinoma samples (datasets (GDS2609) via the GEO database (https://www.ncbi.nlm.nih.gov/geo), *LKB1*, liver kinase B1; *NFKB1*, Nuclear factor NF–kappa–B p105 subunit; *TRAF4*, TNF receptor–associated factor 4; *ITGAV*, Integrin alpha–V.

Using PICRUSt, we identified 14,860 KEGG orthologs (KOs) and 446 pathways from 566 participants. Among these, 37 orthologs and 8 pathways showed significant differences between the OSCC and Control groups. Subsequently, we performed fold change analysis, Wilcoxon tests, AUC evaluation, and logistic regression for each ortholog and pathway to confirm these findings (Tables S3–S5). Pathways such as PI3K–Akt signaling, general pathways in cancer, and nicotinate and nicotinamide metabolism exhibited significantly higher activity in the OSCC group than that in the Control group. Increased activity in these pathways showed a positive correlation with *Rhodococcus* abundance, as indicated by Spearman’s rank correlation coefficients (Figure S1A–B). Parallel analyzes revealed high correlations between *Rhodococcus* and *Slackia* compared with other microbiome components (Figure S1C–D). Among the identified orthologs, liver kinase B1 (LKB1), nuclear factor NF–kappa–B p105 subunit (NFkB1), integrin alpha–V (ITGAV), and TNF receptor–associated factor 4 (TRAF4) exhibited significantly higher abundance in the OSCC group (Figure 5A–G). Additionally, box plots also show significant upregulation of these orthologs in the OSCC group compared with that in the Control group (Figure 5H–K), suggesting their potential involvement in host immune regulation and cancer–related signaling. These findings highlight potential microbial biomarkers and therapeutic targets for OSCC in never–smoking females.

## Discussion

This study is the first to comprehensively characterize both the taxonomic and functional profiles of the salivary microbiome in never–smoking female patients with OSCC. The findings revealed significant alterations in the oral microbiome of patients with OSCC compared with that in healthy controls, with increased abundances of *Rhodococcus*, *Slackia*, *Lactobacillus*, and *Enterobacterales_g*, alongside a notable decrease in *Corynebacterium*. While previous studies have demonstrated microbial compositional shifts in non–smoking patients with OSCC, they did not exclusively focus on never–smokers nor stratify by gender [25]. Consequently, the findings of these studies may reflect broader heterogeneity in exposure histories or biological background [26,27]. In contrast, our findings highlight distinct microbiome profiles in never–smoking female patients with OSCC, significantly expanding upon prior research [28]. Our cohort demonstrated substantial enrichments in taxa such as *Rhodococcus* (30.9-fold increase), *Slackia* (4.73-fold increase), *Lactobacillus* (109.64-fold increase), and *Enterobacterales_g* (45.57-fold increase). Among these taxa, the marked enrichment of *Rhodococcus* is particularly intriguing due to its limited documentation in oral cancer microbiome studies, suggesting a potentially unique carcinogenic mechanism specific to never–smoking females. Although *Rhodococcus* is typically considered an environmental or opportunistic pathogen, it has been detected in tumor–associated microbiota in cancers such as papillary thyroid carcinoma [29]. However, its association with cancer based on oral microbiome profiling has not been previously reported. This enrichment in never–smoking female patients with OSCC may reflect a shift in the mucosal niche favoring pro–tumorigenic bacteria in the absence of conventional carcinogenic exposures [30].

Functional pathway inference using PICRUSt revealed significant upregulation of the PI3K–Akt signaling pathway, nicotinate and nicotinamide metabolism, and other cancer–associated KEGG pathways in the never–smoking female OSCC group [31]. These pathways showed positive correlations with *Rhodococcus* abundance, suggesting a potential mechanistic link between specific microbial taxa and oncogenic processes. The PI3K–Akt pathway is a central regulator of cell growth, metabolism, and survival, and its hyperactivation has been well documented in diverse cancers [32]. It promotes angiogenesis, resistance to apoptosis, and enhanced tumor invasiveness [32,33]. Although *Rhodococcus* is not traditionally classified as an oncogenic microbiome member, certain species produce immunoactive components such as long–chain mycolic acid glycolipids, which can interact with innate immune receptors, including TLR2 and TLR4 [34]. Activation of these receptors can initiate various intracellular signaling cascades, including the PI3K–Akt pathway [34]. In addition, *Rhodococcus* produces cholesterol oxidase, which is involved in lipid metabolism [35]. Lipid and phospholipid metabolites generated through this process may modulate the local signaling environment to regulate Akt phosphorylation [36]. Beyond PI3K–Akt signaling, our functional profiling also revealed significant enrichment of the nicotinate and nicotinamide metabolism pathway. This pathway is essential for the biosynthesis of NAD^+^, a key coenzyme involved in redox reactions, DNA repair, and cell proliferation and survival [37]. While *Rhodococcus* is broadly recognized for its ability to degrade aromatic compounds, its role in nicotinate metabolism remains to be elucidated [38]. *Rhodococcus* may interact with host–associated metabolic pathways, potentially influencing the availability of microbially derived metabolites that affect host NAD^+^ biosynthesis. Although direct evidence remains limited, such microbe–host metabolic interactions could support a microenvironment conducive to tumor progression [39]. This hypothesis may be particularly relevant in never–smoking females with OSCC, where classical carcinogenic drivers are absent and alternative mechanisms such as immune modulation and metabolic reprogramming likely play more prominent roles.

Our functional profiling analysis further revealed significant associations between microbiome composition and predicted expression levels of several oncogenic orthologs, including nuclear factor *NFKB1*, *ITGAV*, *TRAF4*, and *LKB1*. These molecules are key regulators of cancer–related signaling pathways such as inflammation, immune modulation, and cell adhesion. NF-κB1, a central transcription factor in inflammatory signaling, promotes tumor development by enhancing cell proliferation, inhibiting apoptosis, and facilitating metastasis [40,41]. Chronic activation of NF-κB1 in the tumor microenvironment is associated with a pro–tumorigenic immune milieu, particularly in cancers lacking classical mutagenic triggers [42,43]. ITGAV, a subunit of αv integrins, plays a crucial role in extracellular matrix remodeling and signal transduction, and its overexpression has been linked to epithelial–mesenchymal transition (EMT) and poor prognosis in various malignancies, including head and neck squamous cell carcinoma [44]. Similarly, TRAF4, a member of the TNF receptor–associated factor family, contributes to immune signaling and EMT via the NF-κB and TGF-*β* pathways and is frequently upregulated in epithelial tumors [45]. LKB1 is traditionally characterized as a tumor suppressor due to its activation of the AMPK pathway, which regulates cellular metabolism and energy homeostasis [46]. However, recent studies suggest that under certain stress conditions, such as tumor hypoxia or nutrient deprivation, LKB1 may paradoxically enhance tumor cell survival and metastatic potential by promoting metabolic flexibility [47]. This dual role highlights the contextual complexity of LKB1 signaling. It may be particularly relevant in never–smoking female patients with OSCC, where non–traditional carcinogenic pathways such as immune and metabolic dysregulation could play central roles in tumorigenesis [48].

The correlation between oral microbial dysbiosis and oncogenic ortholog expression suggests that the oral microbiome may influence host cancer–related signaling in never–smoking patients with OSCC. Our integrated taxonomic, functional, and ortholog–level analyzes provide novel system–level insights into tumor–associated microbial dynamics in this underrepresented group. However, the present study is limited by its cross–sectional design, relatively small sample size, and potential confounding factors such as alcohol consumption, antibiotic exposure, diet, and comorbidities. Saliva was selected because it is a non–invasive, easily accessible biofluid that reflects the overall oral microbiome and inflammatory milieu, making it suitable for capturing systemic as well as local changes associated with OSCC. Further studies incorporating gnotobiotic models or epithelial co–culture systems are required to validate the mechanistic role of the microbiome in host tumor signaling.

## Conclusion

Our study demonstrates a significant association between oral microbiome dysbiosis and OSCC in never–smoking females. The identification of distinct microbial signatures and enriched functional pathways suggests that the microbiome may contribute to carcinogenesis in the absence of traditional risk factors. These findings highlight the potential of microbiome–based biomarkers for early detection and support further research into targeted prevention and therapeutic strategies.

## Ethical approval

This study was approved by the National Cancer Center, Korea (institutional review board [IRB] approval numbers NCC2019−0050 and NCC2019−0116), and written informed consent was obtained from all participants.

## Supplementary Material

Supplementary Material **Figure S1.** Correlation of functional profiling analysis Correlation analysis and functional profiling of microbiota and pathways in non-smoking female patients with oral squamous cell carcinoma. (A, B) Correlation analysis between five genera and eight functional pathways in both groups. (C–E) Correlation analysis between five genera and specific orthologues involved in PI3K-Akt signaling pathways and cancer-related pathways.**Figure S2.** Panoramic examples of periodontitis stages 1–4 Representative panoramic radiographs of patients illustrating each stage of periodontitis: a) Stage 1, b) Stage 2, c) Stage 3, and d) Stage 4. These images were selected as representative examples of each stage based on the 2017 classification of periodontal diseases proposed by the World Workshop on the Classification of Periodontal and Peri-Implant Diseases and Conditions (Tonetti et al., J Periodontol, 2018), which considers radiographic bone loss, clinical attachment level, and tooth loss due to periodontitis.**Table S1**. Logistic regression analysis of five genera for oral cancer risks.**Table S2.** Logistic regression analysis of 20 genera for oral cancer risks in the LightGBM model.**Table S3**. Logistic regression analysis of three pathways, four orthologies for oral cancer risks.**Table S4.** Logistic regression analysis of 37 orthologs for oral cancer risk.Table S5. Logistic regression analysis of eight pathways for oral cancer risk.**Table S6.** Radiographic criteria for staging of periodontitis and distribution of 72 never-smoking female patients with oral squamous cell carcinoma (OSCC).

## Data Availability

The data that support the finding of this study are available on the request from the corresponding author (M.K.K).
