# Supplementary material for: Functional profiling of the oral microbiome reveals microbial and oncogenic signatures in never-smoking female patients with oral squamous cell carcinoma
Source: J Oral Microbiol. 2025 Dec 4;17(1):2594842. doi: 10.1080/20002297.2025.2594842 (PMC12679845; doi:10.1080/20002297.2025.2594842)
Supplement: Supplementary Material — Figure S1. Correlation of functional profiling analysis Correlation analysis and functional profiling of microbiota and pathways in non-smoking female patients with oral squamous cell carcinoma. (A, B) Correlation analysis between five genera and eight functional pathways in both groups. (C–E) Correlation analysis between five genera and specific orthologues involved in PI3K-Akt signaling pathways and cancer-related pathways.Figure S2. Panoramic examples of periodontitis stages 1–4 Representative panoramic radiographs of patients illustrating each stage of periodontitis: a) Stage 1, b) Stage 2, c) Stage 3, and d) Stage 4. These images were selected as representative examples of each stage based on the 2017 classification of periodontal diseases proposed by the World Workshop on the Classification of Periodontal and Peri-Implant Diseases and Conditions (Tonetti et al., J Periodontol, 2018), which considers radiographic bone loss, clinical attachment level, and tooth loss due to periodontitis.Table S1. Logistic regression analysis of five genera for oral cancer risks.Table S2. Logistic regression analysis of 20 genera for oral cancer risks in the LightGBM model.Table S3. Logistic regression analysis of three pathways, four orthologies for oral cancer risks.Table S4. Logistic regression analysis of 37 orthologs for oral cancer risk.Table S5. Logistic regression analysis of eight pathways for oral cancer risk.Table S6. Radiographic criteria for staging of periodontitis and distribution of 72 never-smoking female patients with oral squamous cell carcinoma (OSCC). [file ZJOM_A_2594842_SM4942.docx]

**Figure S1.**


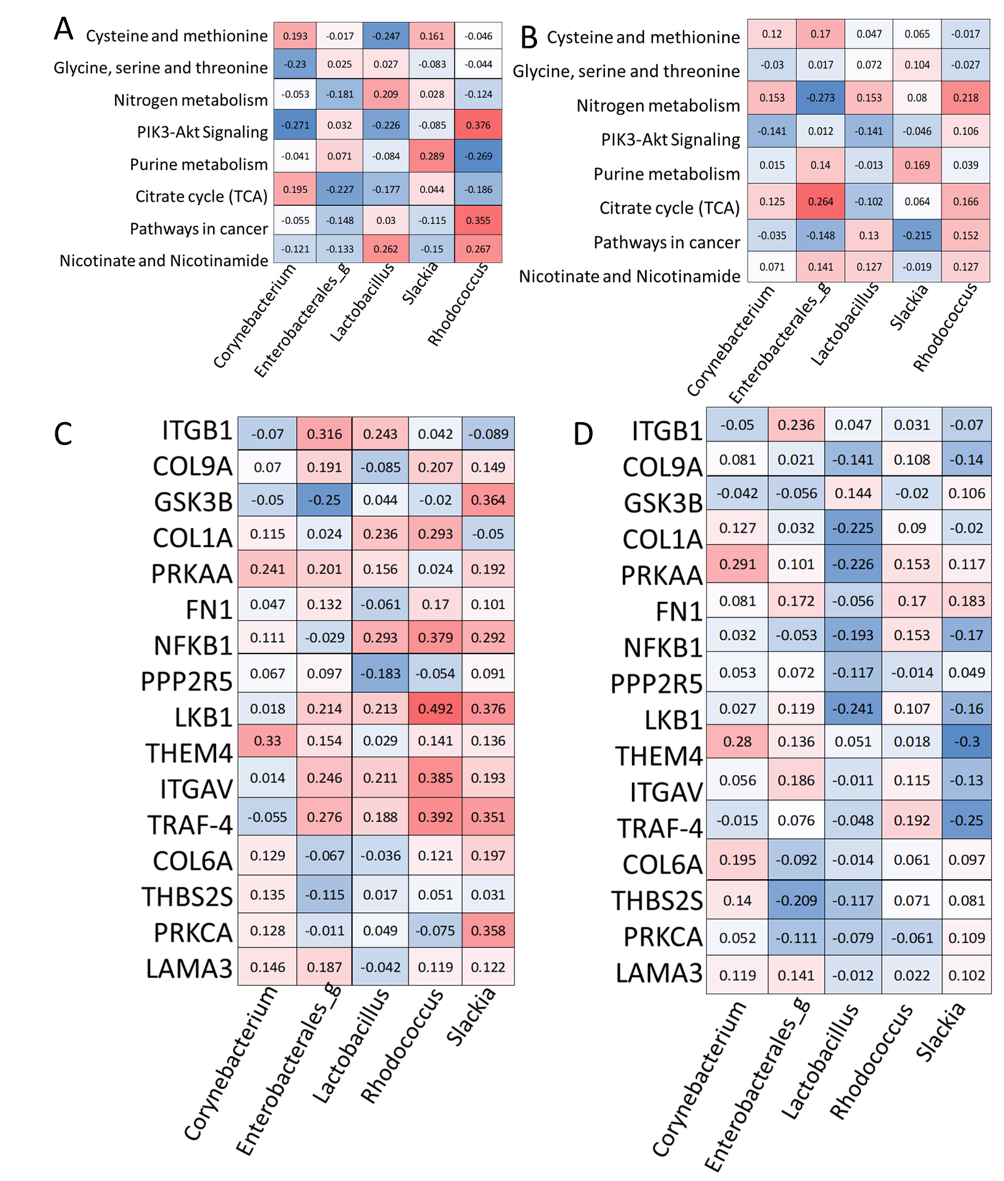


**Figure S2.**


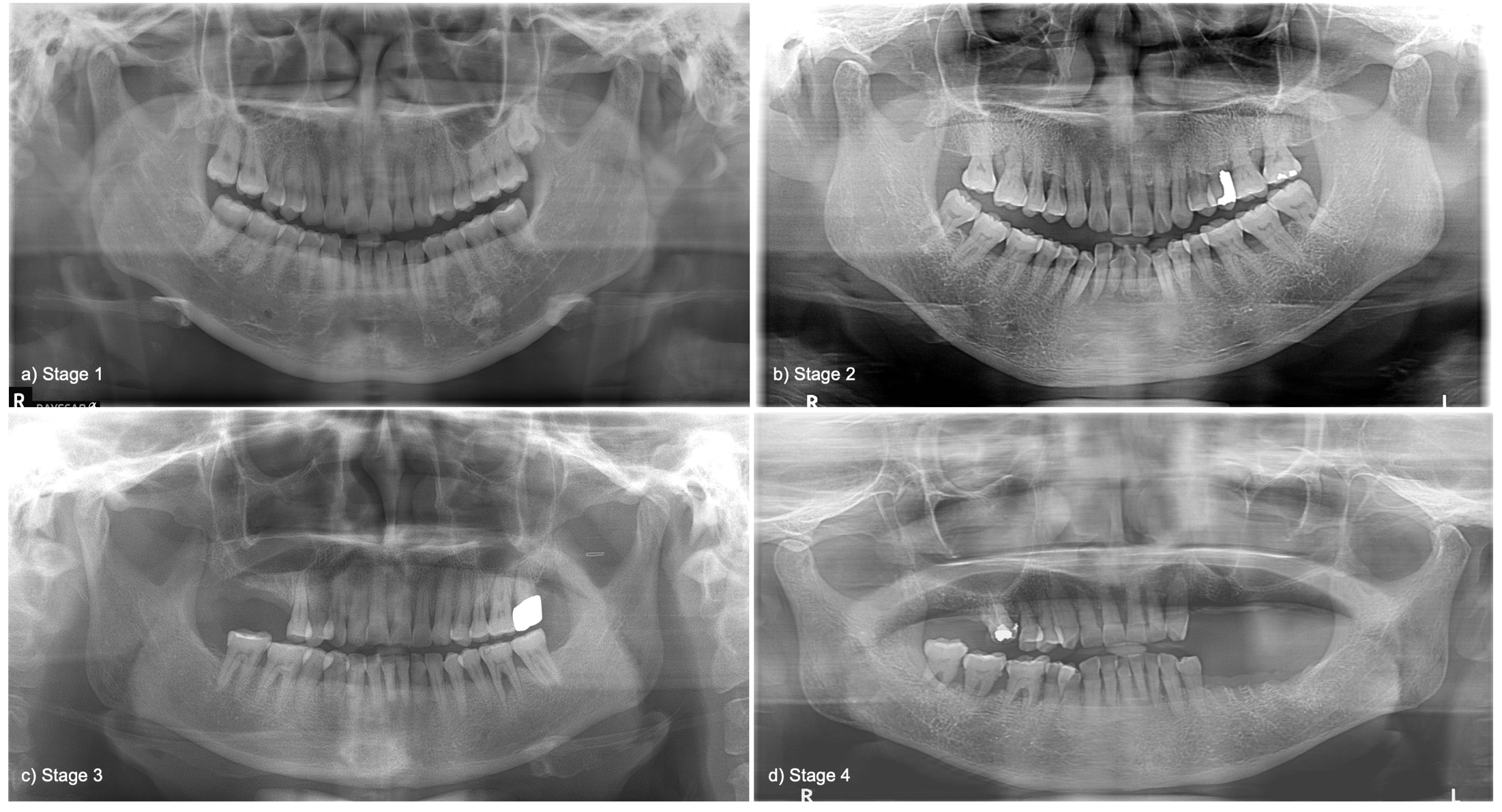


**Table S1**.

| **Microbiome** | **FC** | **Wilcoxon** | **AUC** | **Logistic Regression** | **Univariate OR**  **(95%CI)** | **Univariate *p***^b^ | **Multivariate OR**^a^ **(95%CI)** | **Multivariate *p***^b^ |
| --- | --- | --- | --- | --- | --- | --- | --- | --- |
| Corynebacterium | 0.54 | 1.71E-36 | 0.78 | Continuous scale | 0.08 (0.04~0.31) | 1.55E-04 | 0.07 (0.02~0.29) | 2.34E-04 |
|  |  |  |  | Quartile4 | 0.15 (0.06~0.33) | 2.28E-06 | 0.16 (0.07~0.36) | 7.84E-06 |
|  |  |  |  | Quartile3 | 0.17 (0.08~0.36) | 3.90E-06 | 0.16 (0.07~0.35) | 4.33E-06 |
|  |  |  |  | Quartile2 | 0.13 (0.05~0.30) | 1.75E-06 | 0.13 (0.06~0.32) | 4.08E-06 |
|  |  |  |  | Quartile1 | ref |  | ref |  |
| Enterobacterales_g | 45.57 | 1.08E-22 | 0.78 | Continuous scale | 1.15 (0.89~1.52) | 2.58E-01 | 1.17 (0.91~1.50) | 2.27E-01 |
|  |  |  |  | ≥0 | 8.91 (5.11~15.5) | 1.26E-14 | 7.91 (4.46~14.0) | 1.68E-12 |
|  |  |  |  | <0 | ref |  | ref |  |
| Lactobacillus | 109.6 | 1.58E-27 | 0.80 | Continuous scale | 8.10 (4.80~15.3) | 5.70E-05 | 11.1 (5.81~19.4) | 4.65E-05 |
|  |  |  |  | ≥0 | 8.31 (4.28~16.1) | 3.75E-10 | 7.52 (3.82~14.7) | 5.07E-09 |
|  |  |  |  | <0 | ref |  | ref |  |
| Rhodococcus | 30.9 | 2.71E-37 | 0.65 | Continuous scale | 7.12 (3.10~12.5) | 5.48E-03 | 7.23 (3.58~14.5) | 6.47E-03 |
|  |  |  |  | ≥0 | 36.8 (14.4~94.1) | 5.00E-14 | 56.3 (19.0~96.7) | 3.30E-13 |
|  |  |  |  | <0 | ref |  | ref |  |
| Slackia | 4.73 | 2.34E-10 | 0.68 | Continuous scale | 2.06 (0.58~5.00) | 5.49E-07 | 4.31 (1.58~9.00) | 9.43E-07 |
|  |  |  |  | ≥0 | 2.45 (1.50~4.01) | 3.27E-04 | 2.19 (1.31~3.66) | 2.64E-03 |
|  |  |  |  | <0 | ref |  | ref |  |

Logistic regression analysis was performed with log scaled data. ^a^Multivariate logistic regression was adjusted for sex, age, smoking, drinking and BMI status. ^b^*p* value represents significance of the regression coefficient. OR, Odds ratio; LR, Logistic regression; OC, oral cancer; CI, confidence interval.

**Table S2.**

| **Microbiome** | **FC** | **Wilcoxon** | **AUC** | **Logistic Regression** | **Univariate OR**  **(95%CI)** | **Univariate *p***^b^ | **Multivariate OR**^a^ **(95%CI)** | **Multivariate *p***^b^ |
| --- | --- | --- | --- | --- | --- | --- | --- | --- |
| *Abiotrophia* | 4.65 | 1.37E-03 | 0.59 | Continuous scale | 1.31 (0.61~9.49) | 1.57E-05 | 2.05 (1.05~5.98) | 2.59E-05 |
|  |  |  |  | Quartile4 | 2.05 (1.18~3.55) | 9.94E-03 | 1.67 (0.94~2.97) | 7.99E-02 |
|  |  |  |  | Quartile3 | 0.97 (0.50~1.88) | 9.31E-01 | 0.84 (0.42~1.69) | 6.27E-01 |
|  |  |  |  | Quartile2 | 0.49 (0.06~3.89) | 5.06E-01 | 0.34 (0.04~2.78) | 3.15E-01 |
|  |  |  |  | Quartile1 | ref |  | ref |  |
| *Actinomyces* | 1.35 | 1.71E-01 | 0.51 | Continuous scale | 1.18 (1.02~1.37) | 2.34E-02 | 1.20 (1.08~1.40) | 3.34E-02 |
|  |  |  |  | Quartile4 | 1.08 (0.58~1.99) | 7.96E-01 | 0.93 (0.49~1.78) | 8.33E-01 |
|  |  |  |  | Quartile3 | 0.46 (0.21~0.99) | 4.77E-02 | 0.42 (0.19~0.94) | 3.36E-02 |
|  |  |  |  | Quartile2 | 0.66 (0.33~1.31) | 2.42E-01 | 0.64 (0.31~1.31) | 2.20E-01 |
|  |  |  |  | Quartile1 | ref |  | ref |  |
| *Capnocytophaga* | 1.79 | 4.46E-03 | 0.65 | Continuous scale | 1.31 (1.12~1.53) | 3.43E-04 | 1.34 (1.13~1.55) | 3.31E-04 |
|  |  |  |  | Quartile4 | 3.07 (1.56~6.03) | 1.07E-03 | 2.87 (1.42~5.78) | 3.21E-03 |
|  |  |  |  | Quartile3 | 0.85 (0.36~1.97) | 7.11E-01 | 0.87 (0.36~2.07) | 7.51E-01 |
|  |  |  |  | Quartile2 | 1.00 (0.44~2.26) | 9.84E-01 | 1.05 (0.45~2.40) | 9.16E-01 |
|  |  |  |  | Quartile1 | ref |  | ref |  |
| *Cardiobacterium* | 1.02 | 1.59E-09 | 0.61 | Continuous scale | 1.04 (0.25~3.25) | 9.66E-01 | 1.06 (0.26~4.35) | 9.29E-01 |
|  |  |  |  | Quartile4 | 0.57 (0.32~1.01) | 5.71E-02 | 0.64 (0.35~1.17) | 1.47E-01 |
|  |  |  |  | Quartile3 | 0.22 (0.10~0.48) | 1.44E-04 | 0.24 (0.11~0.52) | 3.63E-04 |
|  |  |  |  | Quartile2 | 0.12 (0.04~0.33) | 2.47E-05 | 0.13 (0.05~0.35) | 5.14E-05 |
|  |  |  |  | Quartile1 | ref |  | ref |  |
| *Corynebacterium* | 0.54 | 1.71E-36 | 0.78 | Continuous scale | 0.08 (0.04~0.31) | 1.55E-04 | 0.07 (0.02~0.29) | 2.34E-04 |
|  |  |  |  | Quartile4 | 0.15 (0.06~0.33) | 2.28E-06 | 0.16 (0.07~0.36) | 7.84E-06 |
|  |  |  |  | Quartile3 | 0.17 (0.08~0.36) | 3.90E-06 | 0.16 (0.07~0.35) | 4.33E-06 |
|  |  |  |  | Quartile2 | 0.13 (0.05~0.30) | 1.75E-06 | 0.13 (0.06~0.32) | 4.08E-06 |
|  |  |  |  | Quartile1 | ref |  | ref |  |
| *Dialister* | 3.89 | 3.43E-05 | 0.65 | Continuous scale | 6.89 (2.89~15.8) | 3.02E-07 | 7.47 (3.69~15.1) | 2.21E-08 |
|  |  |  |  | Quartile4 | 2.64 (1.36~5.13) | 4.09E-03 | 2.39 (1.20~4.76) | 1.31E-02 |
|  |  |  |  | Quartile3 | 0.93 (0.42~2.07) | 8.71E-01 | 0.94 (0.42~2.15) | 8.92E-01 |
|  |  |  |  | Quartile2 | 0.93 (0.42~2.07) | 8.71E-01 | 0.97 (0.43~2.21) | 9.50E-01 |
|  |  |  |  | Quartile1 | ref |  | ref |  |
| *Enterobacterales_g* | 45.57 | 1.08E-22 | 0.78 | Continuous scale | 1.15 (0.89~1.52) | 2.58E-01 | 1.17 (0.91~1.50) | 2.27E-01 |
|  |  |  |  | ≥0 | 8.91 (5.11~15.5) | 1.26E-14 | 7.91 (4.46~14.0) | 1.68E-12 |
|  |  |  |  | <0 | ref |  | ref |  |
| *Eubacterium_g10* | 0.78 | 5.18E-13 | 0.70 | Continuous scale | 0.25 (0.05~1.71) | 1.65E-01 | 0.24 (0.03~1.98) | 1.86E-01 |
|  |  |  |  | Quartile4 | 0.28 (0.14~0.53) | 1.14E-04 | 0.24 (0.12~0.48) | 5.28E-05 |
|  |  |  |  | Quartile3 | 0.10 (0.03~0.26) | 2.53E-06 | 0.10 (0.04~0.27) | 4.41E-06 |
|  |  |  |  | Quartile2 | 0.16 (0.07~0.36) | 6.67E-06 | 0.16 (0.07~0.36) | 9.21E-06 |
|  |  |  |  | Quartile1 | ref |  | ref |  |
| *Haemophilus* | 0.85 | 2.11E-09 | 0.60 | Continuous scale | 0.94 (0.87~1.03) | 4.28E-02 | 0.96 (0.93~1.00) | 4.00E-02 |
|  |  |  |  | Quartile4 | 0.62 (0.34~1.10) | 1.06E-01 | 0.81 (0.44~1.51) | 5.12E-01 |
|  |  |  |  | Quartile3 | 0.21 (0.09~0.48) | 2.04E-04 | 0.26 (0.11~0.59) | 1.37E-03 |
|  |  |  |  | Quartile2 | 0.24 (0.11~0.52) | 3.46E-04 | 0.24 (0.11~0.55) | 5.69E-04 |
|  |  |  |  | Quartile1 | ref |  | ref |  |
| *Lactobacillus* | 109.64 | 1.58E-27 | 0.80 | Continuous scale | 8.10 (4.80~15.3) | 5.70E-05 | 11.1 (5.81~19.4) | 4.65E-05 |
|  |  |  |  | ≥0 | 8.31 (4.28~16.1) | 3.75E-10 | 7.52 (3.82~14.7) | 5.07E-09 |
|  |  |  |  | <0 | ref |  | ref |  |
| *Leuconostoc* | 17.84 | 1.43E-24 | 0.64 | Continuous scale | 8.05 (6.00~24.0) | 3.50E-06 | 11.0 (6.30~19.8) | 6.03E-07 |
|  |  |  |  | ≥0 | 12.7 (6.33~25.5) | 8.75E-13 | 11.2 (5.23~24.0) | 5.38E-10 |
|  |  |  |  | <0 | ref |  | ref |  |
| *Lautropia* | 2.33 | 9.27E-01 | 0.55 | Continuous scale | 1.13 (1.04~1.36) | 6.21E-05 | 1.16 (1.08~1.26) | 7.11E-05 |
|  |  |  |  | Quartile4 | 1.39 (0.77~2.51) | 2.73E-01 | 1.45 (0.77~2.72) | 2.47E-01 |
|  |  |  |  | Quartile3 | 0.52 (0.25~1.10) | 8.93E-02 | 0.53 (0.24~1.15) | 1.07E-01 |
|  |  |  |  | Quartile2 | 0.43 (0.20~0.95) | 3.90E-02 | 0.46 (0.20~1.04) | 6.12E-02 |
|  |  |  |  | Quartile1 | ref |  | ref |  |
| *Olsenella* | 5.32 | 8.01E-09 | 0.65 | Continuous scale | 9.75 (6.05~16.7) | 3.88E-04 | 10.7 (5.05~21.9) | 8.79E-04 |
|  |  |  |  | ≥0 | 2.24 (1.38~3.64) | 1.10E-03 | 2.02 (1.22~3.36) | 6.68E-03 |
|  |  |  |  | <0 | ref |  | ref |  |
| *Oribacterium* | 0.73 | 2.89E-08 | 0.62 | Continuous scale | 0.49 (0.22~1.09) | 3.58E-02 | 0.50 (0.25~0.99) | 4.68E-02 |
|  |  |  |  | Quartile4 | 0.34 (0.16~0.71) | 4.08E-03 | 0.40 (0.19~0.84) | 1.64E-02 |
|  |  |  |  | Quartile3 | 0.66 (0.36~1.21) | 1.80E-01 | 0.72 (0.38~1.36) | 3.15E-01 |
|  |  |  |  | Quartile2 | 0.40 (0.20~0.81) | 1.14E-02 | 0.45 (0.22~0.92) | 2.79E-02 |
|  |  |  |  | Quartile1 | ref |  | ref |  |
| *Parvimonas* | 4.31 | 2.04E-06 | 0.66 | Continuous scale | 5.82 (2.56~12.5) | 5.10E-07 | 6.01 (3.06~11.8) | 2.04E-07 |
|  |  |  |  | Quartile4 | 3.45 (1.68~7.06) | 6.87E-04 | 3.03 (1.44~6.36) | 3.42E-03 |
|  |  |  |  | Quartile3 | 1.37 (0.60~3.11) | 4.45E-01 | 1.38 (0.60~3.18) | 4.51E-01 |
|  |  |  |  | Quartile2 | 1.19 (0.51~2.76) | 6.83E-01 | 1.14 (0.48~2.71) | 7.61E-01 |
|  |  |  |  | Quartile1 | ref |  | ref |  |
| *Selenomonas* | 1.79 | 5.68E-01 | 0.57 | Continuous scale | 1.36 (1.05~1.66) | 6.77E-03 | 1.41 (1.11~1.78) | 4.24E-03 |
|  |  |  |  | Quartile4 | 1.75 (0.95~3.19) | 6.90E-02 | 1.81 (0.96~3.44) | 6.80E-02 |
|  |  |  |  | Quartile3 | 0.45 (0.19~1.03) | 6.05E-02 | 0.41 (0.17~0.96) | 4.11E-02 |
|  |  |  |  | Quartile2 | 0.65 (0.31~1.37) | 2.64E-01 | 0.63 (0.29~1.36) | 2.40E-01 |
|  |  |  |  | Quartile1 | ref |  | ref |  |
| *Veillonella* | 0.75 | 7.20E-07 | 0.64 | Continuous scale | 0.94 (0.91~0.98) | 1.19E-03 | 0.99 (0.87~1.08) | 1.28E-03 |
|  |  |  |  | Quartile4 | 0.21 (0.09~0.49) | 3.65E-04 | 0.23 (0.10~0.55) | 9.69E-04 |
|  |  |  |  | Quartile3 | 0.54 (0.29~1.02) | 6.12E-02 | 0.58 (0.30~1.12) | 1.02E-01 |
|  |  |  |  | Quartile2 | 0.58 (0.31~1.07) | 8.40E-02 | 0.58 (0.30~1.12) | 1.05E-01 |
|  |  |  |  | Quartile1 | ref |  | ref |  |
| *Rhodococcus* | 30.97 | 2.71E-37 | 0.65 | Continuous scale | 7.12 (3.10~12.5) | 5.48E-03 | 7.23 (3.58~14.5) | 6.47E-03 |
|  |  |  |  | ≥0 | 36.8 (14.4~94.1) | 5.00E-14 | 56.3 (19.0~96.7) | 3.30E-13 |
|  |  |  |  | <0 | ref |  | ref |  |
| *Slackia* | 4.73 | 2.34E-10 | 0.68 | Continuous scale | 2.06 (0.58~5.00) | 5.49E-07 | 4.31 (1.58~9.00) | 9.43E-07 |
|  |  |  |  | ≥0 | 2.45 (1.50~4.01) | 3.27E-04 | 2.19 (1.31~3.66) | 2.64E-03 |
|  |  |  |  | <0 | ref |  | ref |  |
| *Stenotrophomonas* | 26.66 | 2.27E-27 | 0.69 | Continuous scale | 3.00 (0.89~9.00) | 2.84E-08 | 3.50 (0.90~11.0) | 2.77E-06 |
|  |  |  |  | ≥0 | 8.28 (4.76~14.4) | 6.69E-14 | 8.00 (4.42~14.4) | 6.06E-12 |
|  |  |  |  | <0 | ref |  | ref |  |

Logistic regression analysis was performed with log scaled data. ^a^Multivariate logistic regression was adjusted for sex, age, smoking, drinking and BMI status. ^b^*p* value represents significance of the regression coefficient. OR, Odds ratio; LR, Logistic regression; OC, oral cancer; CI, confidence interval.

**Table S3**.

| Pathway |  | Name | FC | AUC | Wilcoxon |
| --- | --- | --- | --- | --- | --- |
| ko05200 |  | Pathway in cancer | 2.30 | 0.75 | 1.09E-08 |
|  | K07298 | LKB1; Liver kinase B1 | 4.67 | 0.72 | 2.21E-10 |
|  | K02580 | NFKB1; Nuclear factor NF-kappa-B | 1.52 | 0.61 | 2.78E-03 |
|  | K06487 | ITGAV; Integrin alpha-V is a protein. | 1.31 | 0.59 | 1.36E-02 |
|  | K09848 | TRAF4; TNF receptor-associated factor 4 | 1.50 | 0.57 | 3.92E-02 |
| Ko04151 |  | PI3K-Akt signaling pathway | 2.51 | 0.81 | 6.10E-13 |
|  | K07298 | LKB1; Liver kinase B1 | 4.67 | 0.72 | 2.21E-10 |
|  | K02580 | NFKB1; Nuclear factor NF-kappa-B | 1.52 | 0.61 | 2.78E-03 |
|  | K06487 | ITGAV; Integrin alpha-V is a protein. | 1.31 | 0.59 | 1.36E-02 |
|  | K09848 | TRAF4; TNF receptor-associated factor 4 | 1.50 | 0.57 | 3.92E-02 |
| ko00760 |  | Nicotinate and nicotinamide metabolism | 2.25 | 0.74 | 3.51E-12 |
|  | K07298 | LKB1; Liver kinase B1 | 4.67 | 0.72 | 2.21E-10 |

FC, fold change, AUC, Area under curve, Wilcoxon *p*-value (*p* < 0.05)

**Table S4.**

| **Function** | **FC** | **Wilcoxon** | **AUC** | **Logistic Regression** | **Univariate OR**  **(95%CI)** | **Univariate *p***^b^ | **Multivariate OR**^a^  **(95%CI)** | **Multivariate *p***^b^ |  |
| --- | --- | --- | --- | --- | --- | --- | --- | --- | --- |
| collagen, type IX, alpha(K08131) | 2.57 | 1.12E-09 | 0.72 | Continuous scale | 2.37 (1.80~3.12) | 0.000009 | 2.54 (1.50~3.68) | 0.000008 |  |
|  |  |  |  | Quartile4 | 3.92 (2.03~7.57) | 0.000047 | 3.22 (1.62~6.39) | 0.000833 |  |
|  |  |  |  | Quartile3 | 0.46 (0.17~1.26) | 0.133332 | 0.40 (0.15~1.11) | 0.079839 |  |
|  |  |  |  | Quartile2 | 0.54 (0.20~1.40) | 0.208511 | 0.50 (0.19~1.31) | 0.158517 |  |
|  |  |  |  | Quartile1 | ref |  | ref |  |  |
| collagen, type I, alpha(K06236) | 1.39 | 7.32E-04 | 0.62 | Continuous scale | 1.45 (1.14~1.86) | 0.003041 | 1.43 (1.16~1.89) | 0.005642 |  |
|  |  |  |  | Quartile4 | 2.64 (1.36~5.13) | 0.004091 | 2.57 (1.28~5.14) | 0.007771 |  |
|  |  |  |  | Quartile3 | 0.72 (0.30~1.68) | 0.448391 | 0.76 (0.32~1.84) | 0.548380 |  |
|  |  |  |  | Quartile2 | 1.15 (0.53~2.46) | 0.714713 | 1.04 (0.47~2.30) | 0.930599 |  |
|  |  |  |  | Quartile1 | ref |  | ref |  |  |
| 5'-AMP-activated protein kinase, catalytic alpha subunit(K07198) | 1.82 | 4.20E-07 | 0.68 | Continuous scale | 1.73 (1.31~2.29) | 0.000102 | 1.72 (1.27~2.30) | 0.000130 |  |
|  |  |  |  | Quartile4 | 4.20 (2.01~8.74) | 0.000125 | 3.24 (1.52~6.92) | 0.002392 |  |
|  |  |  |  | Quartile3 | 1.81 (0.80~4.08) | 0.150477 | 1.51 (0.66~3.50) | 0.331960 |  |
|  |  |  |  | Quartile2 | 0.70 (0.26~1.91) | 0.493428 | 0.60 (0.22~1.67) | 0.326240 |  |
|  |  |  |  | Quartile1 | ref |  | ref |  |  |
| serine/threonine-protein phosphatase 2A regulatory subunit B'(K11584) | | 1.48 | 4.07E-03 | 0.60 | Continuous scale | 1.33 (1.03~1.71) | 0.030820 | 1.34 (1.05~1.73) | 0.031234 |
|  |  |  |  |  | Quartile4 | 2.95 (1.42~6.12) | 0.003547 | 2.55 (1.20~5.46) | 0.015364 |
|  |  |  |  |  | Quartile3 | 1.39 (0.61~3.16) | 0.422476 | 1.43 (0.62~3.30) | 0.406946 |
|  |  |  |  |  | Quartile2 | 1.79 (0.82~3.93) | 0.141894 | 1.88 (0.84~4.21) | 0.123945 |
|  |  |  |  |  | Quartile1 | ref |  | ref |  |
| serine/threonine-protein kinase 11(K07298) | 4.67 | 2.21E-10 | 0.72 | Continuous scale | 3.08 (2.19~4.34) | 0.000001 | 3.10 (2.20~4.36) | 0.000001 |  |
|  |  |  |  | Quartile4 | 5.50 (2.48~12.1) | 0.000025 | 4.48 (1.97~10.1) | 0.000334 |  |
|  |  |  |  | Quartile3 | 1.76 (0.71~4.35) | 0.218253 | 1.47 (0.58~3.73) | 0.414766 |  |
|  |  |  |  | Quartile2 | 1.38 (0.53~3.56) | 0.497890 | 1.35 (0.51~3.56) | 0.539911 |  |
|  |  |  |  | Quartile1 | ref |  | ref |  |  |
| acyl-coenzyme A thioesterase THEM4(K16339) | 1.81 | 1.06E-04 | 0.64 | Continuous scale | 1.55 (1.19~2.01) | 0.001046 | 1.57 (1.21~2.04) | 0.001128 |  |
|  |  |  |  | Quartile4 | 2.84 (1.44~5.61) | 0.002541 | 2.93 (1.43~5.98) | 0.003251 |  |
|  |  |  |  | Quartile3 | 1.08 (0.49~2.40) | 0.839405 | 1.06 (0.46~2.42) | 0.888819 |  |
|  |  |  |  | Quartile2 | 1.00 (0.44~2.26) | 0.984334 | 1.09 (0.47~2.53) | 0.839780 |  |
|  |  |  |  | Quartile1 | ref |  | ref |  |  |
| collagen, type VI, alpha(K06238) | 2.10 | 9.28E-07 | 0.67 | Continuous scale | 1.77 (1.39~2.26) | 0.000046 | 1.77 (1.39~2.26) | 0.000063 |  |
|  |  |  |  | Quartile4 | 4.30 (2.06~8.93) | 0.000093 | 4.25 (1.99~9.06) | 0.000185 |  |
|  |  |  |  | Quartile3 | 1.51 (0.65~3.49) | 0.333484 | 1.91 (0.81~4.54) | 0.141760 |  |
|  |  |  |  | Quartile2 | 0.90 (0.35~2.30) | 0.838348 | 1.03 (0.40~2.68) | 0.948725 |  |
|  |  |  |  | Quartile1 | ref |  | ref |  |  |
| thrombospondin 2/3/4/5(K04659) | 1.81 | 8.25E-06 | 0.66 | Continuous scale | 1.64 (1.27~2.11) | 0.000157 | 1.63 (1.19~2.09) | 0.000495 |  |
|  |  |  |  | Quartile4 | 3.00 (1.52~5.89) | 0.001428 | 2.71 (1.34~5.47) | 0.005471 |  |
|  |  |  |  | Quartile3 | 1.31 (0.61~2.83) | 0.478332 | 1.34 (0.61~2.94) | 0.470601 |  |
|  |  |  |  | Quartile2 | 0.62 (0.24~1.54) | 0.306674 | 0.56 (0.22~1.42) | 0.220129 |  |
|  |  |  |  | Quartile1 | ref |  | ref |  |  |
| classical protein kinase C alpha type(K02677) | 1.63 | 6.05E-06 | 0.66 | Continuous scale | 1.82 (1.38~2.39) | 0.000019 | 1.81 (1.34~2.40) | 0.000077 |  |
|  |  |  |  | Quartile4 | 3.77 (1.73~8.20) | 0.000785 | 3.39 (1.52~7.54) | 0.002772 |  |
|  |  |  |  | Quartile3 | 2.35 (1.03~5.33) | 0.040833 | 1.90 (0.81~4.46) | 0.138128 |  |
|  |  |  |  | Quartile2 | 1.45 (0.60~3.53) | 0.405625 | 1.55 (0.63~3.83) | 0.343587 |  |
|  |  |  |  | Quartile1 | ref |  | ref |  |  |
| laminin, alpha 3/5(K06240) | 1.68 | 1.92E-05 | 0.65 | Continuous scale | 1.63 (1.27~2.10) | 0.000124 | 1.61 (1.22~2.08) | 0.000383 |  |
|  |  |  |  | Quartile4 | 2.85 (1.48~5.51) | 0.001753 | 2.67 (1.34~5.31) | 0.005035 |  |
|  |  |  |  | Quartile3 | 0.93 (0.42~2.07) | 0.870730 | 0.93 (0.41~2.10) | 0.854965 |  |
|  |  |  |  | Quartile2 | 0.72 (0.30~1.68) | 0.448391 | 0.80 (0.33~1.91) | 0.614166 |  |
|  |  |  |  | Quartile1 | ref |  | ref |  |  |
| integrin beta 1(K05719) | 1.48 | 1.60E-04 | 0.63 | Continuous scale | 1.52 (1.22~1.91) | 0.000234 | 1.54 (1.25~1.96) | 0.000134 |  |
|  |  |  |  | Quartile4 | 3.10 (1.45~6.59) | 0.003313 | 2.75 (1.25~6.03) | 0.011526 |  |
|  |  |  |  | Quartile3 | 2.31 (1.05~5.07) | 0.035320 | 2.07 (0.92~4.65) | 0.078654 |  |
|  |  |  |  | Quartile2 | 1.31 (0.55~3.10) | 0.538256 | 1.15 (0.48~2.78) | 0.757490 |  |
|  |  |  |  | Quartile1 | ref |  | ref |  |  |
| glycogen synthase kinase 3 beta(K03083) | 1.26 | 1.32E-02 | 0.59 | Continuous scale | 1.35 (1.05~1.74) | 0.019231 | 1.34 (0.90~1.84) | 0.023870 |  |
|  |  |  |  | Quartile4 | 1.66 (0.88~3.14) | 0.114955 | 1.40 (0.72~2.73) | 0.318093 |  |
|  |  |  |  | Quartile3 | 1.12 (0.56~2.21) | 0.745059 | 0.91 (0.44~1.89) | 0.807963 |  |
|  |  |  |  | Quartile2 | 0.50 (0.21~1.16) | 0.109157 | 0.49 (0.21~1.16) | 0.103480 |  |
|  |  |  |  | Quartile1 | ref |  | ref |  |  |
| fibronectin 1(K05717) | 1.68 | 4.04E-04 | 0.63 | Continuous scale | 1.61 (1.19~2.16) | 0.001823 | 1.60 (1.17~2.20) | 0.001975 |  |
|  |  |  |  | Quartile4 | 2.63 (1.32~5.23) | 0.005589 | 2.68 (1.30~5.51) | 0.007417 |  |
|  |  |  |  | Quartile3 | 1.17 (0.53~2.56) | 0.690317 | 1.21 (0.54~2.74) | 0.644095 |  |
|  |  |  |  | Quartile2 | 1.18 (0.54~2.58) | 0.675389 | 1.12 (0.50~2.52) | 0.776575 |  |
|  |  |  |  | Quartile1 | ref |  | ref |  |  |
| nuclear factor NF-kappa-B p105 subunit(K02580) | 1.52 | 2.78E-03 | 0.61 | Continuous scale | 1.26 (0.98~1.62) | 0.072538 | 1.27 (0.99~1.68) | 0.072538 |  |
|  |  |  |  | Quartile4 | 2.13 (1.10~4.13) | 0.024857 | 2.25 (1.12~4.52) | 0.022376 |  |
|  |  |  |  | Quartile3 | 1.20 (0.58~2.50) | 0.608779 | 1.17 (0.54~2.50) | 0.692588 |  |
|  |  |  |  | Quartile2 | 0.80 (0.36~1.79) | 0.597845 | 0.84 (0.36~1.95) | 0.690000 |  |
|  |  |  |  | Quartile1 | ref |  | ref |  |  |
| integrin alpha V(K06487) | 1.31 | 1.36E-02 | 0.59 | Continuous scale | 1.20 (0.93~1.55) | 0.150864 | 1.21 (0.91~1.53) | 0.174366 |  |
|  |  |  |  | Quartile4 | 2.07 (1.04~4.10) | 0.037106 | 1.78 (0.87~3.66) | 0.114064 |  |
|  |  |  |  | Quartile3 | 1.15 (0.53~2.46) | 0.714713 | 1.15 (0.53~2.54) | 0.720252 |  |
|  |  |  |  | Quartile2 | 1.29 (0.61~2.72) | 0.492986 | 1.19 (0.55~2.58) | 0.667295 |  |
|  |  |  |  | Quartile1 | ref |  | ref |  |  |
| RAC serine/threonine-protein kinase(K04456) | 1.19 | 4.52E-05 | 0.64 | Continuous scale | 1.43 (1.06~1.91) | 0.017414 | 1.44 (1.07~1.94) | 0.027641 |  |
|  |  |  |  | Quartile4 | 4.37 (1.95~9.80) | 0.000340 | 4.30 (1.87~9.89) | 0.000586 |  |
|  |  |  |  | Quartile3 | 2.14 (0.89~5.14) | 0.088472 | 2.23 (0.90~5.52) | 0.081410 |  |
|  |  |  |  | Quartile2 | 2.14 (0.89~5.14) | 0.088472 | 2.10 (0.85~5.18) | 0.108776 |  |
|  |  |  |  | Quartile1 | ref |  | ref |  |  |
| cytochrome c(K08738) | 1.40 | 1.69E-04 | 0.63 | Continuous scale | 1.73 (1.33~2.26) | 0.000057 | 1.75 (1.31~2.40) | 0.000086 |  |
|  |  |  |  | Quartile4 | 3.50 (1.66~7.37) | 0.000990 | 2.80 (1.29~6.06) | 0.009148 |  |
|  |  |  |  | Quartile3 | 1.41 (0.60~3.29) | 0.426330 | 1.35 (0.56~3.25) | 0.500114 |  |
|  |  |  |  | Quartile2 | 1.81 (0.80~4.08) | 0.150477 | 1.38 (0.59~3.22) | 0.457932 |  |
|  |  |  |  | Quartile1 | ref |  | ref |  |  |
| Rho-associated protein kinase 1(K04514) | 1.35 | 2.27E-03 | 0.61 | Continuous scale | 1.32 (1.02~1.71) | 0.037386 | 1.31 (0.97~1.82) | 0.037386 |  |
|  |  |  |  | Quartile4 | 2.42 (1.24~4.74) | 0.009476 | 2.12 (1.05~4.27) | 0.036499 |  |
|  |  |  |  | Quartile3 | 0.93 (0.42~2.07) | 0.870730 | 0.93 (0.41~2.12) | 0.871631 |  |
|  |  |  |  | Quartile2 | 1.15 (0.53~2.46) | 0.714713 | 1.14 (0.52~2.49) | 0.737406 |  |
|  |  |  |  | Quartile1 | ref |  | ref |  |  |
| transcription factor E2F3(K06620) | 1.78 | 1.01E-04 | 0.64 | Continuous scale | 1.60 (1.15~2.52) | 0.000432 | 1.62 (1.24~2.12) | 0.000354 |  |
|  |  |  |  | Quartile4 | 2.57 (1.32~5.00) | 0.005419 | 2.29 (1.15~4.59) | 0.019124 |  |
|  |  |  |  | Quartile3 | 1.08 (0.50~2.33) | 0.844407 | 1.07 (0.48~2.36) | 0.875902 |  |
|  |  |  |  | Quartile2 | 0.86 (0.38~1.94) | 0.723909 | 0.93 (0.40~2.13) | 0.854548 |  |
|  |  |  |  | Quartile1 | ref |  | ref |  |  |
| phosphoinositide-3-kinase regulatory subunit alpha/beta/delta(K02649) | 1.83 | 1.05E-03 | 0.62 | Continuous scale | 1.47 (1.15~1.89) | 0.002282 | 1.46 (1.13~1.97) | 0.002569 |  |
|  |  |  |  | Quartile4 | 2.23 (1.19~4.17) | 0.011482 | 2.27 (1.18~4.36) | 0.014293 |  |
|  |  |  |  | Quartile3 | 0.65 (0.29~1.44) | 0.294269 | 0.62 (0.27~1.41) | 0.252406 |  |
|  |  |  |  | Quartile2 | 0.65 (0.29~1.44) | 0.294269 | 0.61 (0.27~1.40) | 0.243506 |  |
|  |  |  |  | Quartile1 | ref |  | ref |  |  |
| Rho guanine nucleotide exchange factor 11(K12331) | 1.82 | 2.59E-02 | 0.58 | Continuous scale | 1.23 (0.99~1.61) | 0.109812 | 1.22 (0.96~1.57) | 0.134465 |  |
|  |  |  |  | Quartile4 | 1.52 (0.83~2.78) | 0.171747 | 1.59 (0.84~3.02) | 0.158115 |  |
|  |  |  |  | Quartile3 | 0.81 (0.41~1.62) | 0.561725 | 0.81 (0.39~1.68) | 0.576659 |  |
|  |  |  |  | Quartile2 | 0.33 (0.13~0.81) | 0.016449 | 0.33 (0.13~0.84) | 0.019632 |  |
|  |  |  |  | Quartile1 | ref |  | ref |  |  |
| phospholipase D1/2(K01115) | 2.07 | 6.86E-06 | 0.66 | Continuous scale | 1.69 (1.31~2.18) | 0.000052 | 1.70 (1.33~2.20) | 0.000054 |  |
|  |  |  |  | Quartile4 | 2.75 (1.47~5.13) | 0.001486 | 2.26 (1.17~4.37) | 0.014974 |  |
|  |  |  |  | Quartile3 | 0.63 (0.27~1.44) | 0.274528 | 0.59 (0.25~1.39) | 0.225633 |  |
|  |  |  |  | Quartile2 | 0.44 (0.17~1.10) | 0.082017 | 0.35 (0.13~0.90) | 0.029571 |  |
|  |  |  |  | Quartile1 | ref |  | ref |  |  |
| E3 ubiquitin-protein ligase XIAP(K04725) | 1.76 | 2.30E-09 | 0.71 | Continuous scale | 2.11 (1.64~2.73) | 0.000001 | 2.09 (1.61~2.72) | 0.000001 |  |
|  |  |  |  | Quartile4 | 5.85 (2.53~13.5) | 0.000037 | 5.72 (2.40~13.6) | 0.000085 |  |
|  |  |  |  | Quartile3 | 3.02 (1.24~7.37) | 0.014916 | 3.02 (1.21~7.58) | 0.018301 |  |
| phosphatidylinositol phospholipase C, beta(K05858) |  |  |  | Continuous scale | 1.29 (1.01~1.64) | 0.038974 | 1.28 (1.00~1.63) | 0.037499 |  |
|  |  |  |  | Quartile4 | 2.00 (1.02~3.90) | 0.041950 | 2.15 (1.06~4.34) | 0.032964 |  |
|  | 1.37 | 1.24E-02 | 0.59 | Quartile3 | 1.14 (0.54~2.38) | 0.723317 | 0.94 (0.43~2.04) | 0.867571 |  |
|  |  |  |  | Quartile2 | 1.15 (0.40~3.27) | 0.790442 | 1.08 (0.37~3.16) |  |  |
|  |  |  |  | Quartile1 | ref |  | ref |  |  |
| protein kinase A(K04345) | 2.14 | 4.95E-08 | 0.69 | Continuous scale | 2.07 (1.59~2.68) | 0.000004 | 2.09 (1.61~2.70) | 0.000004 |  |
|  |  |  |  | Quartile4 | 5.62 (2.54~12.4) | 0.000019 | 5.39 (2.39~12.1) | 0.000050 |  |
|  |  |  |  | Quartile3 | 1.26 (0.48~3.29) | 0.637749 | 1.30 (0.49~3.46) | 0.598987 |  |
|  |  |  |  | Quartile2 | 1.76 (0.71~4.35) | 0.218253 | 1.57 (0.62~3.96) | 0.335948 |  |
|  |  |  |  | Quartile1 | ref |  | ref |  |  |
| DNA polymerase kappa(K03511) | 1.59 | 8.50E-05 | 0.64 | Continuous scale | 1.72 (1.29~2.31) | 0.000264 | 1.73 (1.30~2.34) | 0.000264 |  |
|  |  |  |  | Quartile4 | 2.25 (1.18~4.26) | 0.012919 | 2.24 (1.14~4.40) | 0.018977 |  |
|  |  |  |  | Quartile3 | 1.07 (0.51~2.21) | 0.852981 | 1.20 (0.56~2.58) | 0.633593 |  |
|  |  |  |  | Quartile2 | 0.50 (0.20~1.22) | 0.129049 | 0.56 (0.23~1.40) | 0.216033 |  |
|  |  |  |  | Quartile1 | ref |  | ref |  |  |
| suppressor of fused(K06229) | 2.19 | 8.36E-22 | 0.84 | Continuous scale | 3.92 (2.82~5.45) | 0.000004 | 3.90 (2.80~5.44) | 0.000004 |  |
|  |  |  |  | Quartile4 | 20.0 (6.10~65.4) | 0.000739 | 18.5 (5.61~61.5) | 0.000002 |  |
|  |  |  |  | Quartile3 | 4.03 (1.11~14.6) | 0.034058 | 3.98 (1.08~14.6) | 0.037861 |  |
|  |  |  |  | Quartile2 | 0.67 (0.11~4.09) | 0.666377 | 0.73 (0.12~4.49) | 0.734441 |  |
|  |  |  |  | Quartile1 | ref |  | ref |  |  |
| glutathione S-transferase(K00799) | 1.15 | 4.92E-04 | 0.62 | Continuous scale | 1.57 (1.18~2.09) | 0.002021 | 1.58 (1.20~2.11) | 0.002274 |  |
|  |  |  |  | Quartile4 | 2.33 (1.13~4.79) | 0.021192 | 2.23 (1.05~4.71) | 0.036316 |  |
|  |  |  |  | Quartile3 | 1.93 (0.92~4.05) | 0.081542 | 1.88 (0.87~4.07) | 0.108361 |  |
|  |  |  |  | Quartile2 | 1.17 (0.52~2.64) | 0.694758 | 1.18 (0.51~2.73) | 0.700909 |  |
|  |  |  |  | Quartile1 | ref |  | ref |  |  |
| proto-oncogene serine/threonine-protein kinase Pim-1(K04702) | 1.30 | 2.29E-02 | 0.58 | Continuous scale | 1.03 (0.80~1.31) | 0.833629 | 1.06 (0.79~1.33) | 0.975648 |  |
|  |  |  |  | Quartile4 | 1.54 (0.85~2.79) | 0.148992 | 1.86 (0.99~3.50) | 0.054987 |  |
|  |  |  |  | Quartile3 | 0.73 (0.36~1.46) | 0.378413 | 0.87 (0.42~1.81) | 0.715850 |  |
|  |  |  |  | Quartile2 | 0.22 (0.08~0.62) | 0.003968 | 0.27 (0.10~0.77) | 0.013787 |  |
|  |  |  |  | Quartile1 | ref |  | ref |  |  |
| breast cancer 2 susceptibility protein(K08775) | 2.39 | 8.38E-04 | 0.61 | Continuous scale | 1.59 (1.25~2.01) | 0.000155 | 1.60 (1.27~2.04) | 0.000153 |  |
|  |  |  |  | Quartile4 | 2.51 (1.44~4.39) | 0.001187 | 2.06 (1.15~3.70) | 0.015144 |  |
|  |  |  |  | Quartile3 | 1.01 (0.50~2.02) | 0.965646 | 0.90 (0.44~1.86) | 0.776686 |  |
|  |  |  |  | Quartile2 | 1.11 (0.31~3.96) | 0.866529 | 0.84 (0.22~3.14) | 0.791220 |  |
|  |  |  |  | Quartile1 | ref |  | ref |  |  |
| axin 1(K02157) | 1.37 | 1.04E-04 | 0.64 | Continuous scale | 1.53 (1.19~1.96) | 0.000834 | 1.55 (1.20~1.99) | 0.000129 |  |
|  |  |  |  | Quartile4 | 3.20 (1.50~6.79) | 0.002447 | 3.32 (1.52~7.27) | 0.002649 |  |
|  |  |  |  | Quartile3 | 2.31 (1.05~5.07) | 0.035320 | 2.10 (0.93~4.72) | 0.072860 |  |
|  |  |  |  | Quartile2 | 1.20 (0.50~2.90) | 0.669895 | 1.08 (0.44~2.65) | 0.869989 |  |
|  |  |  |  | Quartile1 | ref |  | ref |  |  |
| histone deacetylase ½(K06067) | 1.46 | 1.03E-02 | 0.59 | Continuous scale | 1.16 (0.90~1.49) | 0.264301 | 1.18 (0.91~1.53) | 0.267512 |  |
|  |  |  |  | Quartile4 | 2.46 (1.23~4.91) | 0.010620 | 2.14 (1.04~4.41) | 0.038643 |  |
|  |  |  |  | Quartile3 | 1.24 (0.57~2.68) | 0.584459 | 1.32 (0.59~2.93) | 0.498722 |  |
|  |  |  |  | Quartile2 | 1.24 (0.57~2.68) | 0.584459 | 1.26 (0.57~2.79) | 0.565310 |  |
|  |  |  |  | Quartile1 | ref |  | ref |  |  |
| TNF receptor-associated factor 4(K09848) | 1.50 | 3.92E-02 | 0.57 | Continuous scale | 1.19 (0.93~1.52) | 0.171667 | 1.17 (0.91~1.54) | 0.143215 |  |
|  |  |  |  | Quartile4 | 1.83 (0.98~3.42) | 0.057696 | 2.00 (1.03~3.90) | 0.040845 |  |
|  |  |  |  | Quartile3 | 0.95 (0.46~1.93) | 0.892001 | 1.00 (0.48~2.11) | 0.993922 |  |
|  |  |  |  | Quartile2 | 0.50 (0.21~1.16) | 0.109157 | 0.56 (0.23~1.34) | 0.192440 |  |
|  |  |  |  | Quartile1 | ref |  | ref |  |  |
| Calmodulin(K02183) | 1.38 | 4.15E-03 | 0.60 | Continuous scale | 1.13 (0.87~1.46) | 0.363861 | 1.12 (0.86~1.46) | 0.106807 |  |
|  |  |  |  | Quartile4 | 1.89 (1.03~3.48) | 0.039699 | 2.45 (1.27~4.73) | 0.007561 |  |
|  |  |  |  | Quartile3 | 0.63 (0.29~1.36) | 0.247150 | 0.76 (0.34~1.70) | 0.501036 |  |
|  |  |  |  | Quartile2 | 0.53 (0.23~1.18) | 0.122972 | 0.66 (0.28~1.54) | 0.338183 |  |
|  |  |  |  | Quartile1 | ref |  | ref |  |  |
| serine/threonine-protein kinase Mtor(K07203) | 1.30 | 3.89E-02 | 0.57 | Continuous scale | 1.34 (1.04~1.71) | 0.021048 | 1.36 (1.10~1.75) | 0.022171 |  |
|  |  |  |  | Quartile4 | 1.75 (0.86~3.55) | 0.117104 | 1.63 (0.78~3.40) | 0.194269 |  |
|  |  |  |  | Quartile3 | 1.56 (0.75~3.20) | 0.225389 | 1.53 (0.72~3.25) | 0.269741 |  |
|  |  |  |  | Quartile2 | 1.34 (0.64~2.83) | 0.428230 | 1.51 (0.70~3.28) | 0.292956 |  |
|  |  |  |  | Quartile1 | ref |  | ref |  |  |
| guanine nucleotide-binding protein G(i) subunit alpha(K04630) | 1.68 | 2.55E-05 | 0.65 | Continuous scale | 1.63 (1.27~2.09) | 0.000140 | 1.62 (1.25~2.05) | 0.000333 |  |
|  |  |  |  | Quartile4 | 2.88 (1.49~5.55) | 0.001610 | 2.68 (1.35~5.32) | 0.004927 |  |
|  |  |  |  | Quartile3 | 0.94 (0.42~2.08) | 0.886323 | 0.93 (0.41~2.11) | 0.867318 |  |
|  |  |  |  | Quartile2 | 0.73 (0.31~1.71) | 0.471090 | 0.81 (0.34~1.93) | 0.628187 |  |
|  |  |  |  | Quartile1 | ref |  | ref |  |  |
| NAD(P)H dehydrogenase (quinone)(K00355) | 1.15 | 3.61E-04 | 0.63 | Continuous scale | 1.65 (1.22~2.23) | 0.001267 | 1.66 (1.23~2.26) | 0.002453 |  |
|  |  |  |  | Quartile4 | 3.25 (1.62~6.50) | 0.000861 | 4.26 (2.05~8.87) | 0.000104 |  |
|  |  |  |  | Quartile3 | 0.84 (0.35~2.01) | 0.696508 | 0.94 (0.38~2.31) | 0.890593 |  |
|  |  |  |  | Quartile2 | 1.34 (0.61~2.95) | 0.462458 | 1.38 (0.61~3.14) | 0.443612 |  |
|  |  |  |  | Quartile1 | ref |  | ref |  |  |

^a^*p* value was computed using wilcoxon rank-sum test for continuous variables. ^b^Quartiles of each genus were divided based on the distribution among oral cancer and controls only. ^c^*p* value was computed using chi-sqaure test for continuous scale and quartiles. OR, odds ratio; CI, confidence interval; OC, oral cancer.

**Table S5.**

| **Function** | **FC** | **Wilcoxon** | **AUC** | **Logistic Regression** | **Univariate OR**  **(95%CI)** | **Univariate *p***^b^ | **Multivariate OR**^a^  **(95%CI)** | **Multivariate *p***^b^ |  |
| --- | --- | --- | --- | --- | --- | --- | --- | --- | --- |
| PI3K-Akt signaling pathway, ko04151 | 2.51 | 6.10E-13 | 0.81 | Continuous scale | 1.34 (1.04~1.73) | 0.023667 | 1.36 (1.08~1.78) | 0.023430 |  |
|  |  |  |  | Quartile4 | 1.76 (0.92~3.36) | 0.084341 | 1.80 (0.92~3.53) | 0.087953 |  |
|  |  |  |  | Quartile3 | 1.00 (0.49~2.06) | 0.982342 | 1.10 (0.52~2.33) | 0.800374 |  |
|  |  |  |  | Quartile2 | 0.77 (0.35~1.65) | 0.504489 | 0.77 (0.35~1.71) | 0.523612 |  |
|  |  |  |  | Quartile1 | ref |  | ref |  |  |
| Pathways in cancer, ko05200 | 2.30 | 1.09E-08 | 0.75 | Continuous scale | 1.99 (1.51~2.63) | 0.000001 | 2.15 (1.59~2.76) | 0.000009 |  |
|  |  |  |  | Quartile4 | 4.25 (1.89~9.54) | 0.000459 | 4.07 (1.77~9.34) | 0.000922 |  |
|  |  |  |  | Quartile3 | 3.02 (1.30~6.99) | 0.009652 | 2.96 (1.25~6.99) | 0.013634 |  |
|  |  |  |  | Quartile2 | 1.38 (0.53~3.56) | 0.497890 | 1.23 (0.47~3.26) | 0.671456 |  |
|  |  |  |  | Quartile1 | ref |  | ref |  |  |
| Glycine, serine and threonine metabolism, ko00260 | 0.99 | 2.22E-02 | 0.58 | Continuous scale | 0.74 (0.58~0.93) | 0.011266 | 0.73 (0.55~0.94) | 0.010231 |  |
|  |  |  |  | Quartile4 | 0.50 (0.25~0.97) | 0.041950 | 0.41 (0.20~0.83) | 0.012916 |  |
|  |  |  |  | Quartile3 | 0.53 (0.27~1.03) | 0.063753 | 0.50 (0.25~1.00) | 0.051189 |  |
|  |  |  |  | Quartile2 | 0.53 (0.27~1.03) | 0.063753 | 0.53 (0.26~1.06) | 0.071017 |  |
|  |  |  |  | Quartile1 | ref |  | ref |  |  |
| Nitrogen metabolism, ko00910 | | 0.98 | 7.77E-04 | 0.62 | Continuous scale | 0.61 (0.47~0.78) | 0.000898 | 0.64 (0.48~0.82) | 0.000099 |
|  |  |  |  |  | Quartile4 | 0.29 (0.13~0.63) | 0.001956 | 0.28 (0.12~0.62) | 0.001960 |
|  |  |  |  |  | Quartile3 | 0.55 (0.29~1.05) | 0.070368 | 0.50 (0.25~0.97) | 0.041884 |
|  |  |  |  |  | Quartile2 | 0.65 (0.35~1.20) | 0.170398 | 0.58 (0.30~1.12) | 0.103478 |
|  |  |  |  |  | Quartile1 | ref |  | ref |  |
| Nicotinate and nicotinamide metabolism, ko00760 | 2.25 | 3.51E-12 | 0.74 | Continuous scale | 0.52 (0.41~0.68) | 0.000068 | 0.56 (0.40~0.72) | 0.000007 |  |
|  |  |  |  | Quartile4 | 0.25 (0.11~0.55) | 0.000580 | 0.25 (0.11~0.56) | 0.000722 |  |
|  |  |  |  | Quartile3 | 0.34 (0.17~0.69) | 0.002996 | 0.35 (0.17~0.72) | 0.004254 |  |
|  |  |  |  | Quartile2 | 0.60 (0.33~1.09) | 0.098130 | 0.52 (0.27~0.98) | 0.044771 |  |
|  |  |  |  | Quartile1 | ref |  | ref |  |  |
| Citrate cycle (TCA cycle), ko00020 | 0.95 | 5.84E-10 | 0.72 | Continuous scale | 0.54 (0.42~0.77) | 0.000002 | 0.52 (0.42~0.66) | 0.000003 |  |
|  |  |  |  | Quartile4 | 0.20 (0.09~0.42) | 0.000017 | 0.18 (0.08~0.37) | 0.000006 |  |
|  |  |  |  | Quartile3 | 0.10 (0.03~0.26) | 0.000029 | 0.10 (0.04~0.26) | 0.000003 |  |
|  |  |  |  | Quartile2 | 0.26 (0.13~0.51) | 0.000908 | 0.23 (0.11~0.46) | 0.000036 |  |
|  |  |  |  | Quartile1 | ref |  | ref |  |  |
| Purine metabolism, ko00230 | 0.97 | 1.55E-05 | 0.65 | Continuous scale | 0.53 (0.41~0.69) | 0.000012 | 0.56 (0.41~0.74) | 0.000001 |  |
|  |  |  |  | Quartile4 | 0.28 (0.14~0.59) | 0.000687 | 0.33 (0.16~0.69) | 0.003261 |  |
|  |  |  |  | Quartile3 | 0.37 (0.19~0.71) | 0.003344 | 0.32 (0.16~0.64) | 0.001421 |  |
|  |  |  |  | Quartile2 | 0.37 (0.19~0.71) | 0.003344 | 0.37 (0.19~0.75) | 0.005382 |  |
|  |  |  |  | Quartile1 | ref |  | ref |  |  |
| Cysteine and methionine metabolism, ko00270 | 0.98 | 1.73E-02 | 0.58 | Continuous scale | 0.72 (0.56~0.93) | 0.011126 | 0.76 (0.57~0.99) | 0.019056 |  |
|  |  |  |  | Quartile4 | 0.44 (0.20~0.93) | 0.032274 | 0.44 (0.20~0.96) | 0.037881 |  |
|  |  |  |  | Quartile3 | 0.76 (0.40~1.46) | 0.419457 | 0.62 (0.31~1.25) | 0.182881 |  |
|  |  |  |  | Quartile2 | 0.88 (0.47~1.65) | 0.707248 | 0.91 (0.47~1.76) | 0.789248 |  |
|  |  |  |  | Quartile1 | ref |  | ref |  |  |

^a^*p* value was computed using Wilcoxon rank-sum test for continuous variables. ^b^Quartiles of each genus were divided based on the distribution among oral cancer and controls only. ^c^*p* value was computed using chi-square test for continuous scale and quartiles. OR, odds ratio; CI, confidence interval; OSCC, oral cancer.

**Table S6**

| Periodontitis stage | Criteria (Tonetti et al., 2017) | Number of patients (n) |
| --- | --- | --- |
| Stage I | Radiographic bone loss <15%; | 20 |
| Stage II | Radiographic bone loss 15–33%; | 7 |
| Stage III | Extending to middle/apical third of root; tooth loss ≤4 | 16 |
| Stage IV | Extending to middle/apical third of root; tooth loss ≥5 | 25 |
| Excluded | No panoramic radiograph | 4 |

**Supplementary Figure**

**Figure S1.** **Correlation of functional profiling analysis** Correlation analysis and functional profiling of microbiota and pathways in non-smoking female patients with oral squamous cell carcinoma. (A, B) Correlation analysis between five genera and eight functional pathways in both groups. (C–E) Correlation analysis between five genera and specific orthologues involved in PI3K-Akt signaling pathways and cancer-related pathways.

**Figure S2.** **Panoramic examples of periodontitis stages 1–4** Representative panoramic radiographs of patients illustrating each stage of periodontitis: a) Stage 1, b) Stage 2, c) Stage 3, and d) Stage 4. These images were selected as representative examples of each stage based on the 2017 classification of periodontal diseases proposed by the World Workshop on the Classification of Periodontal and Peri-Implant Diseases and Conditions (Tonetti et al., *J Periodontol*, 2018), which considers radiographic bone loss, clinical attachment level, and tooth loss due to periodontitis.

**Supplementary Tables**

**Table S1**. Logistic regression analysis of five genera for oral cancer risks.

**Table S2.** Logistic regression analysis of 20 genera for oral cancer risks in the LightGBM model.

**Table S3**. Logistic regression analysis of three pathways, four orthologies for oral cancer risks.

**Table S4.** Logistic regression analysis of 37 orthologs for oral cancer risk.

**Table S5.** Logistic regression analysis of eight pathways for oral cancer risk.

**Table S6.** Radiographic criteria for staging of periodontitis and distribution of 72 never-smoking female patients with oral squamous cell carcinoma (OSCC).
